# Brief report: Therapeutic benefit of ISA-2011B in colorectal cancer

**DOI:** 10.1007/s11033-026-12495-2

**Published:** 2026-08-08

**Authors:** Veroniaina Hanitrarimalala, Jenny Persson, Anette Gjörloff Wingren

**Affiliations:** 1 Department of Biomedical Sciences, Faculty of Health and Society, Malmö University, Malmö, Sweden; 2 Biofilms-Research Center for Biointerfaces, Malmö University, Malmö, Sweden; 3 Department of Molecular Biology, Umeå University, Umeå, Sweden; 4 Department of Bioanalysis, Faculty of Natural Sciences, Kristianstad University, Kristianstad, Sweden

**Keywords:** Colorectal cancer, ISA-2011B, PIP5K1α, Viability, 3D spheroids

## Abstract

ISA-2011B is a phosphatidylinositol-4-phosphate 5-kinase-α (PIP5K1α) inhibitor that has been reported to be selective in suppressing the growth of prostate, breast and hepatic cancer cells. Here, cell viability of 2-dimensional (2D) cultures and 3-dimensional (3D) spheroids of four colorectal cancer (CRC) cell lines with different mutations were evaluated after treatment with the drug ISA-2011B. Methods: The CRC cell lines were investigated for viability in 2D and 3D spheroid cultures. The 3D spheroids were subjected to imaging, and the average volumes were measured. Results: The results show different treatment effects of ISA-2011B on the four CRC cell lines, indicating that mutated Kirsten rat sarcoma viral oncogene homolog (KRAS) and phosphatidylinositol 3-kinase (PI3K) genes can play a role in the outcome of treatment. Conclusions: With additional studies, we suggest that ISA-2011B can be a promising drug target in CRC.

## Introduction

The phosphoinositide lipid family includes numerous derivatives of phosphatidyl inositols (PtdIns) that are generated by a process of phosphorylation by enzymes known as phosphatidylinositol-phosphate kinases (PIPKs) [1]. Phosphatidylinositol 3-kinases (PI3K) are the most well-studied PIPKs and are frequently used as anticancer therapeutic targets [2]. Phosphatidylinositol-4-phosphate 5-kinase-α (PIP5K1α) is a lipid kinase that functions similarly to PI3K. The role of PIP5K1α in oncogenic processes is increasingly recognized as pro-tumorigenic and hence the development of specific inhibitors for PIP5K1α is an emerging field of interest.

ISA-2011B, a new anticancer drug, was discovered by conducting research on heterocyclic compounds. Semenas et al. demonstrated that ISA-2011B showed a high binding affinity for the enzyme PIP5K1α, which suppressed invasion of prostate cancer cells in vitro and prostate cancer tumor growth in vivo [3]. The treatment decreases invasiveness, triggers apoptosis, and blocks tumor growth of prostate cancer cells in xenograft mice. ISA-2011B inhibits PIP5K1α, resulting in reduced PI3K/AKT levels, sustained p27 and down-regulation of cell cycle regulators such as cyclin-dependent kinase 1 (CDK1) [3]. Sarwar et al. also found that ISA-2011B suppresses tumor growth of triple-negative breast cancer (TNBC) cells in a xenograft mouse model [4]. The reduction of growth is likely due to the ability of ISA-2011B to block PIP5K1α and the downstream signals in the elevated PI3K/AKT pathway. Moreover, ISA-2011B treatment lowered the percentage of MDA-MB-231 cells in synthesis (S) phase and Gap 2/mitosis (G2/M) phases, demonstrating its influence on DNA synthesis and cancer cell survival [4]. The association between PIP5K and the reactive oxygen species (ROS)–autophagy–Nrf2 pathway was examined in hepatocellular carcinoma (HCC) tissue samples and hepatic cancer cell lines showing that PIP5K isoforms promote hepatic cancer cell proliferation under oxidative stress conditions [5]. Importantly, targeting PIP5K sensitizes cells to ROS-mediated apoptosis through modulation of the PI3K/AKT/Mammalian Target of Rapamycin (mTOR) and autophagy pathways. Recently, in another study, targeting PIP5K1A using ISA-2011B showed that this could be a promising approach for inhibiting HCC progression and overcoming resistance to ferroptosis inducers, such as the multi-tyrosine kinase inhibitor sorafenib [6]. Thus, PIP5K1α has excellent potential as a therapeutic target, and the ISA-2011B compound is promising for future targeted cancer therapy.

In this study, we investigated the treatment effects of different concentrations of ISA-2011B on four different CRC cell lines in two distinct culture methods in vitro. Our results reveal that ISA-2011B have effect on the growth of the CRC cells, which is assumed to be regulated via the PIP5K1α pathway.

## Materials and methods

### Cell lines and culture

The four different CRC cell lines HCT116, HT29, SW620 and NCI-H508 were obtained from The American Type Culture Collection (ATCC/ Manassas, VA, USA). HCT116 and HT29 cells were cultured in McCoy´s 5 A medium with 10% FBS. SW620 cells, derived from a metastatic CRC cell line from the large intestine, were cultured in Dulbecco´s Modified Eagle Medium (DMEM) with low glucose (1 g/L) and with 10% fetal bovine serum (FBS). NCI-H508, a semi-adherent cell line derived from derived from a metastasis in the abdominal wall, were cultured in Roswell Park Memorial Institute 1640 (RPMI-1640) GlutaMax -I with 10% FBS. The cultures were kept at 37 °C in a humidified atmosphere containing 5% CO2. Medium, FBS and supplements were obtained from Thermo Fisher Scientific, Waltham, USA. For 2-dimensional (2D) cell viability experiments, HCT116, HT29 and SW620 cells were seeded into flat bottom 96-well plates (83.3924.300, Sarstedt, Nümbrecht, Germany) at a cell density of 2,000 cells/well. Cell density of 7,500 cells/well was used for the cell line NCI-H508. Cells were incubated for 24 h to adhere. For 3-dimensional (3D) cell viability assays, cells were seeded into U-bottom 96-well plates (83.3925.500, Sarstedt) at a cell density of 500 cells/well in DMEM F-12 1:1 mixture (Thermo Fisher Scientific) supplemented with B27 (Thermo Fisher Scientific), 20 ng/mL of human epidermal growth factor (hEGF, Sigma-Aldrich, MO, USA) and 20 ng/mL of human basic fibroblast growth factor (bFGF, Sigma-Aldrich). Cells were incubated for hours to build spheroids.

**Fig. 1:**
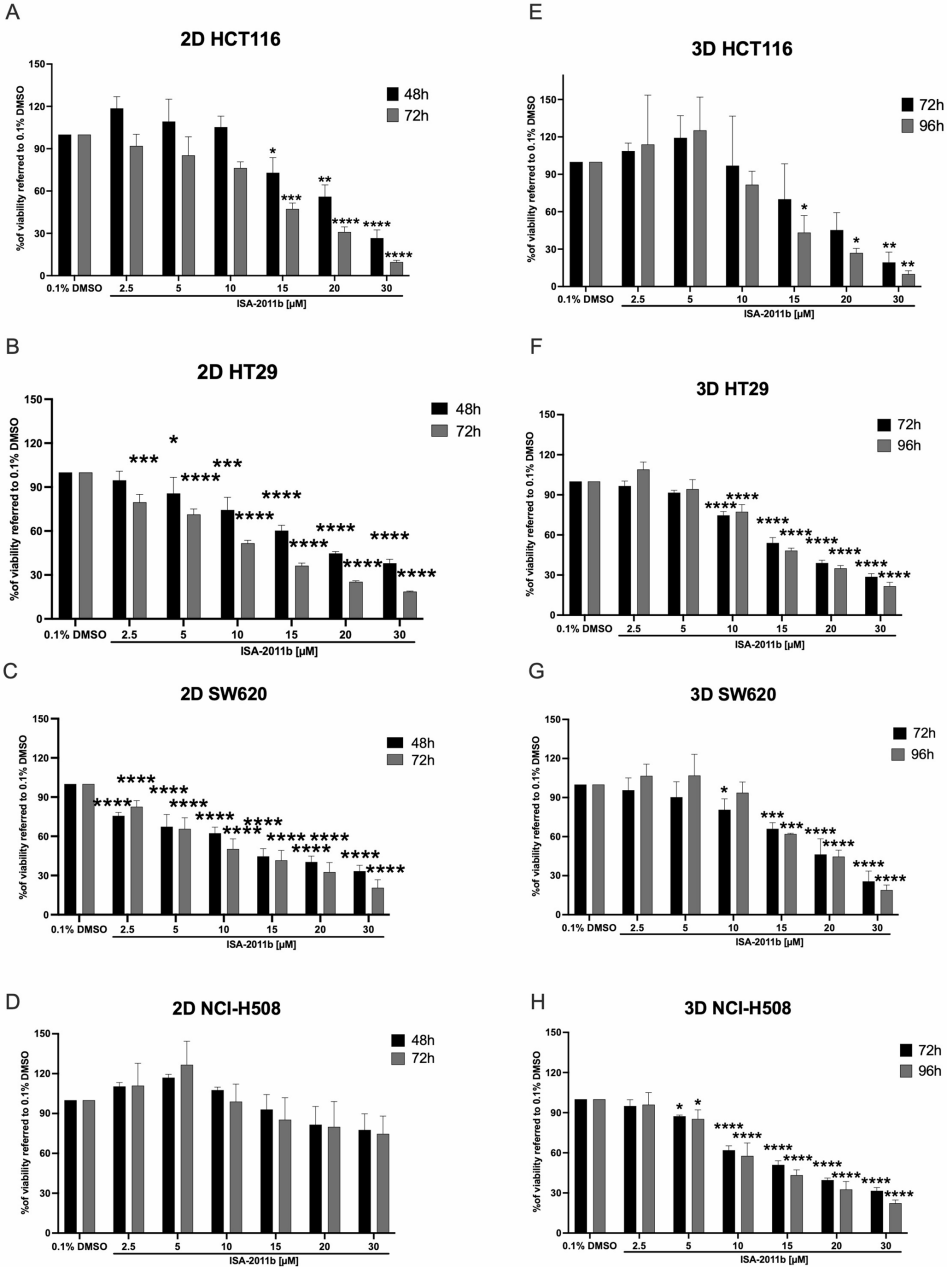
Cell viability in 2D (monolayer) cell culture models of the four CRC cell lines after treatment with ISA-2011B at 48 h and 72 h, respectively (**A**-**D**), and 3D (spheroid) cell culture model of the four CRC cell lines after treatment with ISA-2011B at 72 h and 96 h, respectively (**E**-**H**). Viability was calculated in relation to that of control cells treated with 0.1% DMSO (%). (*n* = 3 in triplicates). * = *p* < 0.05; ** = *p* < 0.01; *** = *p* < 0.005: **** = *p* < 0.001

**Fig. 2:**
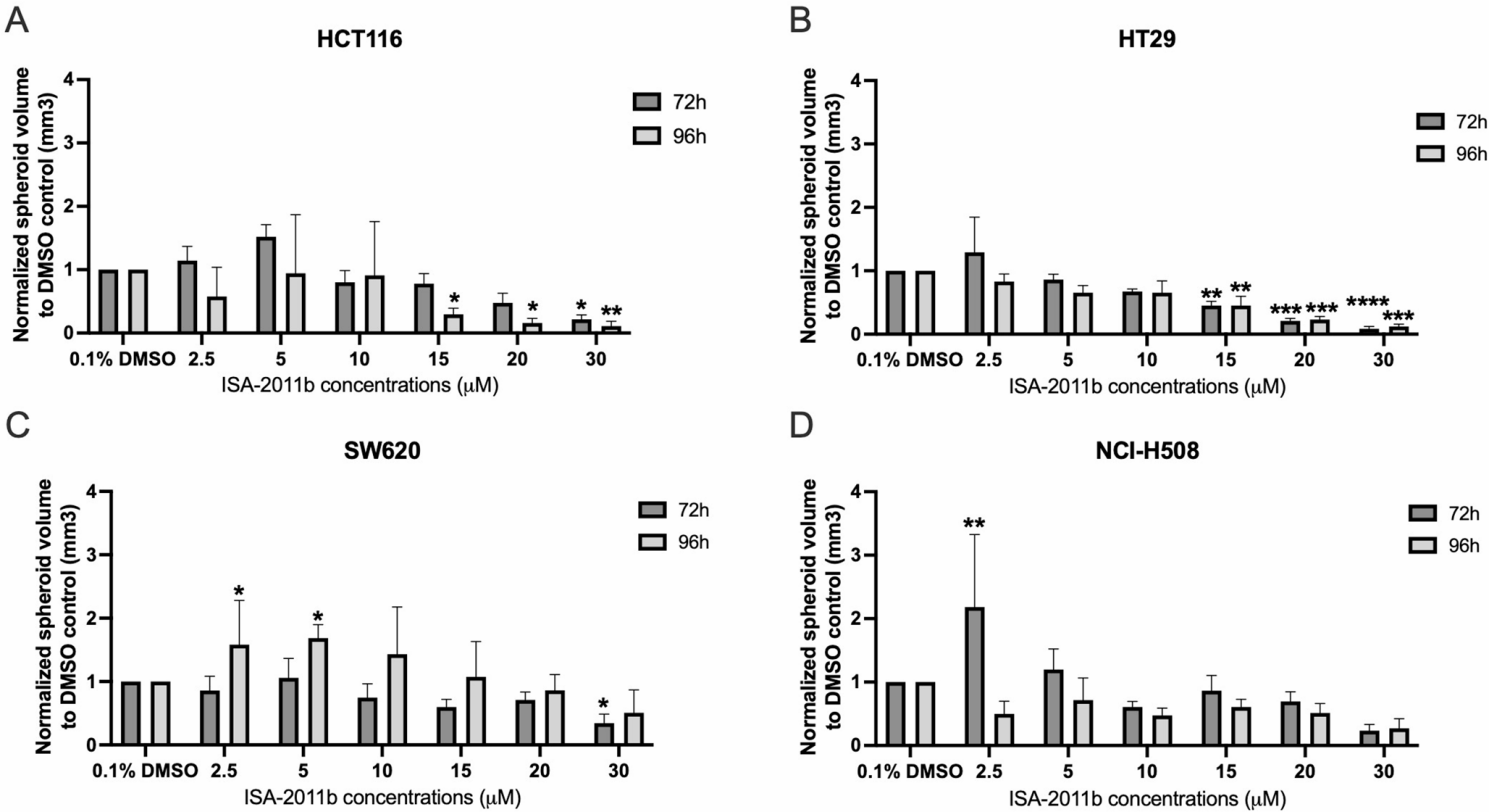
Spheroid volumes of the four CRC cell lines after treatment with ISA-2011B at 72 h and 96 h, respectively (**A**-**D**). Volumes are presented in relation to that of control cells treated with 0.1% DMSO (%), where the control is normalized to 1. (*n* = 3 in triplicates). * = *p* < 0.05; ** = *p* < 0.01; *** = *p* < 0.005: **** = *p* < 0.001

### Drugs

ISA-2011B (Olympia Diagnostics, Sunnyvale, CA, USA), was dissolved in sterile dimethyl sulfoxide (DMSO) (Sigma-Aldrich) and concentrations of 2.5, 5, 10, 15, 20 and 30 µM were tested. The final concentration of DMSO was 0.1%.

### Cell viability measurements

The 3-(4,5-dimethylthiasol-2-yl)-2,5 diphenyltetrazolium bromide (MTT) reduction assay was used for assessing the viability for the 2D cell culture models. Post-treatment 48–72 h, 10 µL MTT-reagent (5 mg/mL in DPBS) (EMD Millipore Corp., USA) was added. After 3 h of incubation, the solution was removed and 100 µL DMSO was added to the attached cells. The absorbance was measured at 570 nm by using SpectraMax^®^ ID3 multi plate reader (Molecular Devices, San Jose, CA, USA). The CellTiter-Glo 3D assay (Promega, Madison, Wisconsin, USA) was used for determining the viability of the 3D cell culture models. Post-treatment 72–96 h, CellTiter-Glo 3D assay was performed according to the manufacturer´s instruction with the exception that the CellTiter-Glo 3D reagent was used in a 1:2 ratio. The luminescence was measured by using SpectraMax^®^ ID3 multi plate reader (Molecular Devices, San Jose, CA, USA). Viability was calculated in relation to control cells treated with 0.1% DMSO alone (shown as %).

### Morphological analysis

The morphology of the spheroids was imaged by using Lumenera Infinity 1 light microscope camera (Ottawa, ON, Canada). The control cells were treated with 0.1% DMSO alone. The spheroid volumes were calculated as described [7], according to the formula V = 4/3 $\:\pi\:$R^3^.

### Statistical data analysis

All data presented are the mean ± standard error of the mean (SEM) from three independent experiments, while for cell viability experiments five technical replicates were carried out. The data was analyzed using 2way ANOVA and Sidak’s test for multiple comparisons. The statistical analyses were performed using Prism 10 (GraphPad Software, San Diego, CA, USA) and a *p*-value of less than 0.05 was considered statistically significant. * = *p* < 0.05; ** = *p* < 0.01; *** = *p* < 0.005: **** = *p* < 0.001.

## Results and discussion

The effect of ISA-2011B on the survival of the four CRC cell lines was evaluated 48 and 72 h after treatment in 2D cultures and 72 and 96 h in 3D spheroid models. In terms of cell viability, all four CRC cell lines showed sensitivity to ISA-2011B (Fig. 1).

For two of the cell lines, addition of higher concentrations of ISA-2011B was necessary for monitoring a reduction in cell viability. This was seen in 2D cultures of NCI-H508 cells and in 3D cultures of HCT116 cells, respectively. The differences between the two culture models are not surprising since studies have shown that the effect of chemotherapeutic compounds in 3D models are distinct from those found in 2D models [8]. The mCRC cell line NCI-H508 is a cell line derived from metastasis to the abdominal wall obtained from a patient after treatment with 5-fluorouracil (5-FU) [9]. A cross-resistance for ISA-2011b that is more pronounced in the 2D model of NCI-H508 cell line could be hypothesized, through for example the p53 status that determines the sensitivity to 5-FU in NCI-H508 cells, where several mutations including a tumor protein 53 (TP53) mutation at R273H has been reported [9].

SW620-derived 3D models formed larger spheroids with various shapes compared to the other cell lines. Higher concentrations of ISA-2011B have an impact on the spheroid volume of all cell lines, at both time points in a dose-dependent manner (Fig. 2).

It has been demonstrated that the disintegration degree of detached cells from CRC 3D spheroids following drug treatment is a useful independent indicator of drug efficacy [10]. Therefore, we can indirectly confirm that HT29 cells are sensitive to ISA-2011B by showing a high statistically significant difference in volumes when comparing control cells with ISA-2011B-treated cells of 20–30 µM concentrations (*p* < 0.001).

Targeting PIP5K1α using ISA-2011B disrupts critical survival and invasive networks. PIP5K1α is situated on chromosome 1q21.3 [11] and synthesizes PtdIns-4,5-P2 (PIP2) which is then utilized by PI3K to generate PtdIns-3,4,5-P3 (PIP3) [12]. Subsequently, PIP3 activates the AKT family of serine/threonine kinases [13] and the phosphorylated AKT at Ser-473 site (pSer-473), which is linked to mutations in PIK3CA (p110α), or the phosphatase and tensin homolog (PTEN) gene in metastatic cancers [14]. In CRC, PI3K pathway mutations occur in roughly 50–70% of patients, with PIK3CA gene changes present in 20% of CRC cases [15]. To our knowledge, the HCT116, HT29 and NCI-H508 cell lines have PIK3CA gene mutations.

PIP5K1α has been exploited as a potential therapeutic target for advanced prostate cancer cells [3] and for castration-resistant prostate cancer (CRPC) cells [16]. Interestingly, it was shown that the N-terminal domain of PIP5K1α was required for mRNA expression and protein stability of PIP5K1α itself. When combining tamoxifen and ISA-2011B treatment, the inhibitory effect on CRPC cells was higher compared to when adding either drug alone [17]. Additionally, ISA-2011B promotes apoptosis with a comparable effect to the drug docetaxel and reduces metastasis in TNBC by blocking the elevated PI3K/AKT pathway [4]. In HCC, growth inhibition [6] and ROS-mediated apoptosis were demonstrated when ISA-2011B targeted the PIP5K isoform [5]. Further work would include mechanistic studies in CRC cells.

NCI-H508 cells are KRAS WT, neuroblastoma RAS viral oncogene homolog (NRAS) WT, but have an inactivating B-Raf proto-oncogene (BRAF) mutation (G596R), a PIK3CA E545K helical domain mutation and a TP53 mutation at R273H [9]. HCT116 cells harbor the KRAS G13D mutant and the PIK3CA mutant H1047R and is the only one out of the four investigated cell lines in this study with a microsatellite instability (MSI) phenotype, caused by various deficiencies in the DNA mismatch-repair system. The SW620 cells carry the KRAS G12V mutant as well as two different TP53 mutations, R273H and P309S. The HT29 cell line is a TP53 R273H mutant but also carries mutations in adenomatous polyposis coli (APC) and BRAF genes.

PIP5K1α indeed acts as a central signaling protein in CRC while promoting tumor growth, survival, invasion, and therapy resistance primarily through phosphoinositide-mediated activation of PI3K/protein kinase B (AKT) and wingless-type MMTV integration site family (Wnt) pathways [18]. KRAS WT tumors may be more dependent on PIP5K1α upstream signaling, making it potentially more impactful as a target [2, 4, 19]. In BRAF-mutant CRC, PIP5K1α acts as a parallel survival pathway enabler, not the primary driver [20]. Moreover, PIP5K1α amplifies survival signaling through AKT in a context where apoptosis is already impaired by TP53 mutations [21]. It has been shown that combined KRAS and TP53 mutations lead to highly aggressive, therapy-resistant tumors. A therapeutic implication could be to target PIP5K1α to exploit reliance on survival signaling in TP53-mutant CRC [22].

## Conclusions

We suggest that the selective PIP5K1α inhibitor, ISA-2011B, inhibits PIP5K1α kinase activity and blocks its downstream PI3K/AKT phosphorylation, leading to reduced cell growth in CRC cells. Taken together, our findings in the present study provide valuable information on novel targets for ISA-2011B including mutated KRAS and PI3K genes, which hold a great potential for additional studies to show development of advanced CRC treatment.

## Data Availability

All data supporting the findings of this study are available within the paper.
